# Digenic Inheritance of Mutations in Homologous Recombination Genes in Cancer Patients

**DOI:** 10.3390/jpm14060584

**Published:** 2024-05-29

**Authors:** Maria Valeria Freire, Marie Martin, Karin Segers, Edith Sepulchre, Natacha Leroi, Jérôme Coupier, Hassan Rezaei Kalantari, Pascal Wolter, Joëlle Collignon, Marc Polus, Olivier Plomteux, Claire Josse, Vincent Bours

**Affiliations:** 1 Department of Human Genetics, GIGA Research Center, University of Liège and CHU Liège, Av. Hippocrate 1/11, 4000 Liège, Belgium; fm216@hotmail.com; 2 Department of Human Genetics, CHU Liège, Domaine Universitaire, 4000 Liège, Belgium; marie.martin@chuliege.be (M.M.); karin.segers@lns.etat.lu (K.S.); e.sepulchre@chuliege.be (E.S.); natacha.leroi@chuliege.be (N.L.);; 3 Onco-Hematology Department, CHR Verviers, Rue du Parc 29, 4800 Verviers, Belgium; hassan_kalantari@hotmail.com; 4 Onco-Hematology Department, St Nikolaus Hospital, Hufengasse 4/8, 4700 Eupen, Belgium; pascal.wolter@klinik.st-vith.be; 5 Department of Medical Oncology, GIGA Research Center, University of Liège and CHU Liège, Domaine Universitaire, 4000 Liège, Belgium; joelle.collignon@chuliege.be (J.C.); c.josse@chuliege.be (C.J.); 6 Department of Gastroenterology, CHU Liège, Av. Hippocrate 1/11, 4000 Liège, Belgium; m.polus@chuliege.be; 7 Gastro-Enterology Department, CHC, Boulevard Patience et Beaujonc 2, 4000 Liège, Belgium; olivier.plomteux@chc.be

**Keywords:** digenic inheritance, double heterozygosity, familial cancer, BRCA1, BRCA2, ATM, CHEK2

## Abstract

Background/Objectives: *BRCA1*, *BRCA2*, *ATM*, and *CHEK2* are known cancer predisposition genes (CPGs), but tumor risk in patients with simultaneous pathogenic variants (PVs) in CPGs remains largely unknown. In this study, we describe six patients from five families with multiple cancers who coinherited a combination of PVs in these genes. Methods: PVs were identified using NGS DNA sequencing and were confirmed by Sanger. Results: Families 1, 2, and 3 presented PVs in *BRCA2* and *ATM*, family 4 in *BRCA2* and *BRCA1*, and family 5 in *BRCA2* and *CHEK2*. PVs were identified using NGS DNA sequencing and were confirmed by Sanger. The first family included patients with kidney, prostate, and breast cancer, in addition to pancreatic adenocarcinomas. In the second family, a female had breast cancer, while a male from the third family had prostate, gastric, and pancreatic cancer. The fourth family included a male with pancreatic cancer, and the fifth family a female with breast cancer. Conclusions: The early age of diagnosis and the development of multiple cancers in the reported patients indicate a very high risk of cancer in double-heterozygous patients associated with PVs in HR-related CPGs. Therefore, in families with patients who differ from other family members in terms of phenotype, age of diagnosis, or type of cancer, the cascade testing needs to include the study of other CPGs.

## 1. Introduction

Cancer predisposition syndromes (CPS) are now extensively studied, with an increasing proportion of cancer patients undergoing genetic testing [1]. This testing is based on the type of cancer, the number of cancer occurrences during the patient’s life, the age at diagnosis, and the family history [2,3]. It is expected that 3 to 5% of cancers are linked to a causal variant in a cancer predisposition gene (CPG) [4]. As most CPS are transmitted in an autosomal dominant way, once a pathogenic variant (PV) is identified in a family, the geneticists propose a family cascade testing to search for the variant, and start with first-degree relatives [5].

Within the frame of inherited cancer predisposition, carriers of pathogenic variants (PVs) in a single gene have been extensively represented in the literature, and an ever-growing accumulation of data on the single gene-related cancer risk, based on multiple family histories, is available [6,7]. These data have led to gene-specific screening and follow-up recommendations for these carriers [3]. However, the coinheritance of heterozygous PV in two CPGs is a poorly studied event restricted to small case series and single case reports [8,9,10]. The exact frequency of double heterozygotes remains unknown, as is the case for their cancer risk and associated follow-up strategies [11]. Therefore, empirically, most genetic centers propose to apply the guidelines defined for the most dangerous gene to the follow-up of patients with two PVs in two different CPGs. However, the *BRCA1* and *BRCA2* PVs coinheritance, in the population-based Israeli national breast cancer cohort, was described in 2.2% of all carriers [12], and 17 double heterozygotes for CPGs were detected in a breast cancer cohort of people of Slavic ancestry which included 5391 patients [13].

Breast cancer gene 1 (*BRCA1*), breast cancer gene 2 (*BRCA2*), checkpoint kinase 2 (*CHEK2*), and ataxia-telangiectasia mutated (*ATM*) are CPGs, part of the homologous recombination (HR) pathway for double strand break (DSB) repair. This pathway preferentially uses the sister chromatid for error-free repair, and both the DNA damage response and the cell cycle checkpoints are crucial for initiating and regulating HR [14]. ATM participates in HR initiation and phosphorylation of CHEK2; BRCA1 facilitates DNA end resection [15], while BRCA2 aids in the formation of a DNA D-loop through the invasion of the nearby duplex DNA [16]. Finally, the BRCA2 protein is post-translationally modified by ATM [17].

HR is crucial for repairing severe replication lesions at replication forks, and can repair or bypass DNA lesions remaining due to inactivation of other pathways. Consequently, mutations in HR genes result in genomic instability, fueling further mutations that lead to cancer development [14,18]. This deficiency in the HR pathway makes tumor cells more sensible to poly-(ADP-ribose)-polymerase inhibitors, platinum derivatives, alkylating agents, mitomycin C, and other antitumor drugs that are used for the treatment of cancer patients [19,20,21,22].

PVs in *BRCA1*, *BRCA2*, *CHEK2*, and *ATM* have been linked to a wide variety of cancers [15]. *BRCA1* and *BRCA2’s* PVs were associated with breast cancer, ovarian/fallopian cancer, pancreas cancer, prostate cancer, and melanoma, while breast, prostate, thyroid, kidney, colon and stomach cancers were related to PVs in *CHEK2* [23]. Germline heterozygous PVs in *ATM* increase the risks of breast, pancreatic, gastro-esophageal, colorectal, ovarian, prostate, thyroid, gastric, and head and neck cancers, as well as melanoma [24]. Given the frequencies of PVs in these genes, it is expected that cancer patients carrying two PVs should be rarely, but not exceptionally, observed. Moreover, as these genes act on the homologous recombination pathway, these double heterozygote patients might have a higher risk of HR dysfunction and thus a more severe cancer risk.

In this study we describe six patients from five families with multiple cancers who coinherited PVs in *BRCA2* and other HR genes—four patients with variants in *BRCA2* and *ATM*, one patient with *BRCA2* and *BRCA1*, and one patient with *BRCA2* and *CHEK2* PVs.

## 2. Materials and Methods

### 2.1. Ethical Approval

The study was conducted in accordance with the Declaration of Helsinki and approved by the Comité d’Ethique Hospitalo-facultaire Universitaire de Liège (protocol code 2019/245 and date of approval 28 October 2019).

### 2.2. Data Collection

Patient sex, age, age at diagnosis for each tumor, and personal and family history were extracted from the medical records. Data on cancer diagnosis and treatment were gathered from the institution’s database. All of the patients read and signed an informed-consent document.

### 2.3. Genetic Analysis

Genetic analysis was performed on DNA extracted from blood samples using QIAcube (QIAGEN, Hilden, Germany) and STARlet (Seegene Inc., Seoul, South Korea) extraction instruments (See Supplementary Data: DNA extraction methods). DNA purity and concentration were measured with NanoDrop (Thermo Fisher Scientific, Waltham, Massachusetts, United States), and DNA underwent NGS panel sequencing (See Supplementary Data: Table S1). The bioinformatic analysis was performed using in-house demultiplexing pipelines and the in-house Humanomics pipeline (as described in [25]). Variant classification was performed according to the ACMG “Standards and guidelines for the interpretation of sequence variants” [26]. The in silico analysis of missense and splicing variants was performed using the aggregated score of the Franklin by Genoox tool (https://franklin.genoox.com, accessed on 17 May 2024), which includes the scores of SIFT, FATHMM, DANN, MetaLR, REVEL, MutationAssessor, PolyPhen-2, MutationTaster, PrimateAI, BayesDel, SpliceAI, dbscSNV, GERP, GenoCanyon, fitCons, MitoTip, and APOGEE. For the splicing variants, Human Splicing Finder [27] was used. Two databases, gnomAD (https://gnomad.broadinstitute.org/, accessed on accessed on 17 May 2024) and ALFA (https://www.ncbi.nlm.nih.gov/snp/docs/gsr/alfa, accessed on accessed on 17 May 2024), were used to retrieve the Minor Allele Frequency (MAF) data. The identified PVs were confirmed by Sanger sequencing (See Supplementary Materials: Table S2).

## 3. Results

### 3.1. Frequency of Double Heterozygotes

Over the past 28 months, following the introduction of the new Hereditary Breast and Ovarian Cancer (HBOC) panel at our institution, a total of 2152 panels have been conducted in cases of cancer patients (1929 13-gene panels and 223 26-gene panels). In total, 121/1929 13-gene panels (6.27%) and 22/223 (9.8%) 26-gene panels were positive, containing a pathogenic or likely pathogenic result. Three patients (3/2152 patients, 0.14%) were double-heterozygous for CPG PVs. Two samples had two PVs in the 13-gene panel (2.2% of the 91 samples with PVs) and one in the 26-gene panel (5.6% of the 18 samples with PVs, see Table 1). Heterozygous variants in genes associated with a recessive instance of CPS, such as the *MUTYH* gene, were excluded from this analysis.

In this study, we report two of the three double-heterozygous patients from whom we obtained informed consent, and one additional patient whose double-heterozygous state was diagnosed based on family history. The three additional included patients were previously observed by the genetics department and/or had a relevant family history.

### 3.2. Clinical History

Six patients from five families underwent genetic consultation in the context of multiple cancers or early-onset disease, leading to the identification of two heterozygous PVs in the HR genes of each patient (see Table 2).

**Figure 1:**
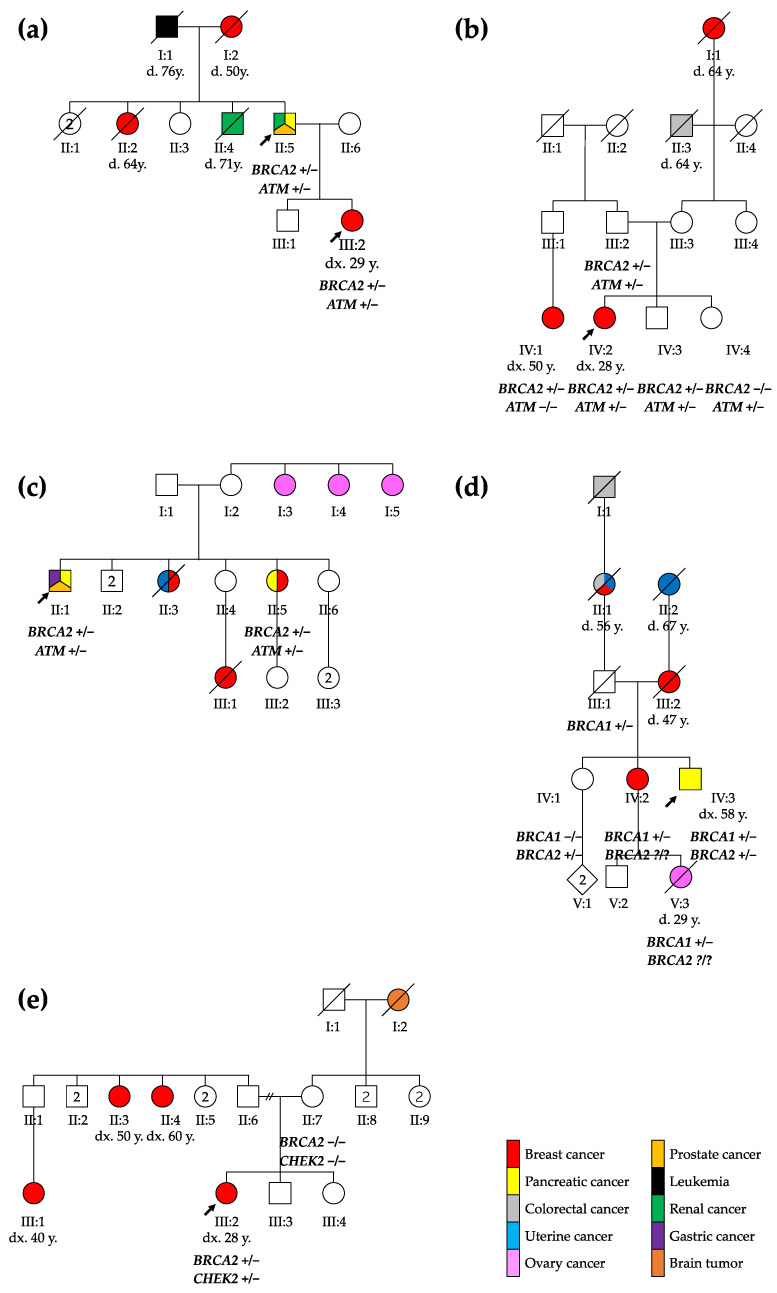
Pedigree of the five families reported: (**a**) family 1, (**b**) family 2, (**c**) family 3, (**d**) family 4, and (**e**) family 5. The probands are marked with arrows. ca., cancer; CRC, colorectal cancer; d., death; dx., diagnosis; y., years; +/−, heterozygous genotype; −/−, homozygous wild type genotype; ?/?, unknown genotype.

The first family included a 67-year-old male with a medical history of multiple cancers whose daughter had been diagnosed with breast cancer (see Figure 1). The male patient presented kidney and prostate cancer and pancreatic adenocarcinoma at the ages of 50, 51, and 66, respectively.

The patient’s daughter was diagnosed with breast cancer at 29 years. A tumorectomy showed grade 3 invasive ductal carcinoma with axillary and mediastinal lymph node extension (ypT1cN2aMx). Two years after the diagnosis, she presented a first relapse with one successfully treated bone metastasis. The subsequent relapses included liver metastasis, lymph node invasion, and finally brain metastasis in 2021.

In the second family, a 28-year-old female underwent an exploratory laparoscopy due to persistent non-specific abdominal pain with nausea and vomiting, showing endometriotic lesions and multiple hepatic lesions described as angiomas. A month later, after a week of hyperthermy and a positive COVID-19 test, the thoracoabdominal computed tomography scan demonstrated a large breast lesion with a highly suspicious right axillary lymph node, necrotic hepatic and bone lesions, and possibly-COVID-19-related pulmonary foci. A grade 3 infiltrating ductal carcinoma was diagnosed and treated.

A 65-year-old male from the third family was diagnosed with a Gleason 3 pT2bNxM0 prostate adenocarcinoma at the age of 49, well-differentiated pT1N0M0 enteric adenocarcinoma one year later, and finally metastatic pancreatic cancer. His older sister was first diagnosed with breast cancer at 60, and then pancreatic cancer at 70 years old.

The fourth family included a 58-year-old male who presented a 15 kg weight loss, fatigue, nausea, and transfixing abdominal pain for 2 weeks. In a tomography, an isthmus pancreatic mass of 4 cm infiltrating peripancreatic fat with hepatic metastasis was discovered (CTxNxM1). In this patient, a familial *BRCA1* variant was found 15 years earlier at the time of a breast cancer diagnosis for his sister at the age of 35 (she developed a second breast cancer 15 years later, and pancreatic cancer at the age of 67). The male patient was known to carry this familial BRCA1 variant, inherited from their father. As the *BRCA1* familial variant was not sufficient to explain both pancreatic cancers, those in the patient and his sister, as well as their mother’s breast cancer, we re-initiated a CPG analysis and this showed that he carried two pathogenic variants: the known familial *BRCA1* PV, and a *BRCA2* PV.

The 29-year-old female from the fifth family discovered three mobile, not painful masses in her right breast while performing self-palpation. The biopsy of one of the masses revealed a ductal breast adenocarcinoma (cT2N0M0). After a right mastectomy with sentinel ganglion, an infiltrating tubular adenocarcinoma (pT2mN1mi) was diagnosed. During genetic evaluation, a *BRCA2* and a *CHEK2* PV were identified in the patient. Both PVs were absent in the mother, while the father was not available for testing. The patient has two sisters, one of whom is underage and has not been tested.

### 3.3. Genetic Characteristics

In the patients from families 1 to 3, genetic analyses showed *BRCA2* and *ATM* PVs. The patients from family 4 and 5 carried PVs in *BRCA1*/*BRCA2* and *BRCA2*/*CHEK2*, respectively.

Three of the identified *BRCA2* nonsense variants were located in exon 11/27 (c.3865_3868del, c.5057T>A, c.4284dup), while the fourth was located in exon 7/27 (c.537dup), leading to the existence of a severely truncated or absent protein due to nonsense-mediated mRNA decay (NMD) [28]. *BRCA2* c.3865_3868del, c.5057T>A, and c.537dup variants were absent from the gnomAD (v2.1.1) and ALFA databases, while *BRCA2* c.4284dup had a frequency of 1 out of 244426 alleles in the total population of gnomAD (v2.1.1) and was absent from the ALFA database (see Table 3). *BRCA2* c.8243G>A had a frequency of 2/249060 in the total population of gnomAD (v2.1.1) and 1/25340 in ALFA. Various functional studies show a loss of function and/or protein stability linked to the *BRCA2* c.8243G>A variant [29,30]. All of the *BRCA2* variants were previously described as pathogenic [22,31,32,33].

The missense *ATM* c.8494C>T variant was located in exon 58 out of 63, was present in 7 out 236730 alleles in the total population in gnomAD (v2.1.1), and has been previously described as pathogenic and associated with an increased cancer risk [34]. The *ATM* c.7516-2A>G variant located in intron 50 out of 62 has not been previously reported, and was not present in the gnomAD (v2.1.1) or ALFA databases. However, the variant was located in a region of the gene where other variants have been described as pathogenic, affecting a conserved splice site [35]. *ATM* c.7516-2A>G in silico evaluation results showed splicing alteration by wild-type acceptor site breakage. The nonsense *ATM* c.6326G>A variant in exon 43 out of 63 was predicted to cause loss-of-function by premature protein truncation or NMD. This variant was not found in the gnomAD (v2.1.1) or ALFA databases and has been previously reported as pathogenic [36].

Nonsense *BRCA1* c.1121del variant caused a frameshift with a predicted stop codon two amino acids after the deletion, which could result in loss of normal protein function through protein truncation or NMD. This variant was absent in gnomAD (v2.1.1) or ALFA, but was present in several individuals suffering from breast and/or ovarian cancer [37]. This variant was also known as c.1240delC in the literature.

Missense *CHEK2* c.499G>A variant leads to a substitution of a highly conserved amino acid. This variant was present in the total population of gnomAD (v2.1.1) in 6 out of 251424 alleles, and 3/100662 alleles in ALFA. Additionally, functional analysis showed a loss of function of the protein due to structural instability [38] or phosphorylation anomaly [39]. The in silico analysis of the variant predicted a deleterious effect on the protein, and CHEK2 loss-of-function variants are known to be pathogenic [40].

## 4. Discussion

PVs in *BRCA1*, *BRCA2*, *CHEK2*, and *ATM* increase the lifetime cancer risk of breast cancer [41]. In women carrying *BRCA1* and *BRCA2* PVs, the cumulative risk of breast cancer was 4% before the age of 30 for each gene, and reached 72% for *BRCA1* and 69% for *BRCA2* by age 80 [42]. For *ATM* variants, there was an estimated breast cancer relative risk of 2.8, and the absolute breast cancer risk reached 27% by 80 years. The *CHEK2* breast cancer risk was variable for different PVs. Common *CHEK2* truncating variants conferred a greater than twofold relative risk, while a less common I157T variant was associated with a 1.4-fold risk [43]. Similarly, in a study that included 65,057 women with breast cancer, the age of diagnosis of CHEK2 PV’s carriers was 47.7 years [41]. However, there is a lack of epidemiological data on BC risk in patients carrying PVs in two of these genes. Our study indicates very precocious and even metastatic BC in women with PVs in *BRCA2* and *ATM* (patients 2 and 3) or *BRCA2* and *CHEK2* genes (patient 6), while a previous study evaluating 17 double-heterozygous patients with breast cancers failed to demonstrate a younger age at presentation in this group [13]. A similar trend could be expected when PVs in *BRCA1* are associated with PVs in other CPGs.

The risks of other cancers are also elevated in *BRCA1*-, *BRCA2*-, and *ATM*-variant carriers. *BRCA1* and *BRCA2* PVs confer increased risks of prostate, pancreatic, and ovarian cancers [44], while moderate-to-high risks of pancreatic (OR 4.21), prostate (OR 2.58), and gastric (OR 2.97) cancers were estimated for *ATM*-variant carriers [24]. In our observations, two male patients were treated for a prostate cancer diagnosed at an early age, which might suggest that the *BRCA2*-linked risk is further increased by the presence of the *ATM* PV.

The reported pancreatic cancer risks in *BRCA1* and *BRCA2* carriers by the age of 70 years were 1.16% and 4.1% in men [44]. As BRCA1, BRCA2, and ATM proteins interact in the HR pathway, an additive effect on HR deficiency could be expected, giving a further increased risk of pancreatic cancer, as observed in patients 1, 4, and 5. Indeed, in a recent case report of a female patient carrying two heterozygous pathogenic variants in *BRCA2* and *ATM*, breast cancer was diagnosed at 34 and pancreatic cancer at 48 years [45]. This raises the question of whether the previously-described reported young women with breast cancer (family 1) will need additional monitoring for their pancreatic cancer risk.

Therefore, our observations suggest that patients carrying a PV in BRCA2 plus another HR gene should be carefully monitored for BC, pancreatic cancer, and prostate cancer. However, incomplete penetrance and variability of the age of onset of the disease are also observed in double-heterozygous patients. In the second reported family, the proband’s father also carried both *BRCA2* and *ATM* PVs (see Figure 1) but did not have any history of cancer, indicating that both genetic and non-genetic factors can influence cancer risk in variant carriers [44], while in the third family, the proband’s sister developed cancer at an older age, supporting the variable expressivity of these mutations. Further studies and larger cohorts are thus of course needed to better define the cancer risk associated with having two PVs in HR genes.

PVs in *BRCA1* and *BRCA2* have frequencies of 0.21% and 0.31% in the European population [46], while the frequencies of *ATM* and *CHEK2* PVs reach 1% [47] and 1.4% [48]. These estimations, taken together, and given the scarcity of double-heterozygotes reports, indicate that the prevalence of digenic coinheritance is likely underestimated. Recently, even a patient with breast cancer and concurrent PVs in three cancer-related genes (*BRCA1*, *BRCA2*, and *CHEK2*) has been reported [49]. Therefore, given the high variability of phenotypes within families and between different families, when a cascade testing is performed after the identification of a familial PV, the assessment should not stop at the single known familial PV, at least in individuals with precocious breast, pancreatic, or prostate cancers; in those with multiple cancers; and in cases of cancers that are not frequently associated with the identified PV, as the possibility of co-segregation of another PV should not be neglected.

The size of the genetic panels used for cancer patients’ evaluation has progressively increased in recent years [50]. Consequently, the findings derived from these expanded panels are still in the preliminary stages, and it is impossible to directly compare the new data with previous results from shorter panels. Nonetheless, instances of double mutations are expected to remain relatively rare. After introducing multi-gene panel testing in 2014, by 2023, in the Fox Chase Cancer Center Risk Assessment Program Registry, 70 patients were found to carry at least two PVs in CPGs (excluding biallelic *MUTYH* PVs) [51]. In a review of 55,803 patients screened with a 25-gene hereditary cancer panel, 106 individuals (0.19%) showed PVs or likely pathogenic variants in two or more genes [52], a frequency of double heterozygotes very similar to that observed in the present study.

With the increase in patient numbers and the utilization of larger cohorts for analysis, more robust data will be available soon. Furthermore, the criteria for recommending genetic studies have undergone multiple revisions over time. Only recently has genetic testing for pancreatic and prostate cancer been included as part of the standard practice [53]. Consequently, the reports of larger cohorts of patients with diverse primary tumors will increase the likelihood of identifying cases with double mutations.

The small number of patients, the bias in recruitment, and the inability to evaluate the segregation in all of the families are the main limitations of our study. Additionally, we did not address the associated treatment strategies—platinum-based chemotherapy or PARP inhibitors—and the patients’ responses. With only six patients, we lack the data for meaningful comparisons or response-rate calculations. A larger study involving double-heterozygous patients is necessary to address these questions effectively.

Therefore, in young cancer patients from a family with a single known CPG PV, it could be useful to evaluate other genes to identify the potential transmission of several PVs and double-heterozygous carriers with a specific high cancer risk. Moreover, our data suggest that the surveillance of patients carrying two PVs in HR genes should include at least breast, pancreas, and prostate cancer screening, starting early. From our limited study, we would recommend starting a screening in those patients, at the latest, from the ages of 25, 40, and 50 for breast, prostate and pancreas cancer, respectively.

## 5. Conclusions

In conclusion, the early age of diagnosis and the development of multiple cancers in the reported patients indicate a very high risk of cancer in double-heterozygous patients associated with PVs in HR-related CPGs. Therefore, when a CPG PV is identified in a family, the usual cascade testing needs also to consider a study of other CPGs in patients with specific phenotypes, even distinct from other family members, either based on the age at diagnosis or the type of cancer.

## Figures and Tables

**Table 1:** Double heterozygote statistics in the institution.

	13-Gene Panel		26-Gene Panel	
	Likely Pathogenic (n = 30)	Pathogenic (n = 91)	Likely Pathogenic (n = 4)	Pathogenic (n = 18)
1 variant per sample, n (%)	30 (100.0)	89 (97.8)	4 (100.0)	17 (94.4)
2 variants per sample, n (%)	0 (0.0)	2 (2.2)	0 (0.0)	1 (5.6)

**Table 2:** Characteristics of the patients included in the study.

	Family 1		Family 2	Family 3	Family 4	Family 5
	Patient 1 II:5	Patient 2 III:2	Patient 3 IV:2	Patient 4 II:1	Patient 5 IV:3	Patient 6 III:2
Sex	Male	Female	Female	Male	Male	Female
Age (years)	67	34	28	65	58	29
Medical history	Childhood asthma, gouty arthritis, hypercholesterolemia, and hypertrophic heart disease	None	None	Essential thrombocytopenia	Type 2 diabetes, volvulus	Glucose intolerance
Oncological history	Renal cancer at 50, prostate cancer at 51, and metastatic pancreatic cancer at 66 years	Breast cancer at 29 years (ER positive, PR positive, Ki67 60%, HER2 ++, SISH −)	Metastatic breast cancer at 28 years (ER positive, PR positive, Ki67 40%, HER2 ++ SISH −)	Prostate cancer at 49, gastric cancer at 60, and metastatic pancreatic cancer at 64 years	Metastatic pancreatic cancer at 57 years	Ductal breast cancer at 28 years (ER positive, PR negative, Ki67 50%, HER2 ++, SISH −)
Treatment	Renal cancer–surgery, prostate cancer–brachytherapy, pancreatic cancer–chemotherapy, and targeted therapy	Neoadjuvant chemotherapy, surgery, and radiotherapy	Chemotherapy	Prostate and gastric cancer–surgery, pancreatic cancer–chemotherapy	Chemotherapy	Surgery, adjuvant chemotherapy, radiotherapy, and hormonal therapy
Identified germline heterozygous pathogenic and likely pathogenic variants						
*BRCA2*	c.3865_3868del p.(Lys1289Alafs*3)	c.3865_3868del p.(Lys1289Alafs*3)	c.5057T>A p.(Leu1686*)	c.4284dup p.(Gln1429Serfs*9)	c.8243G>A p.(Gly2748Asp)	c.537dup p(Ile180Tyrfs*3)
*ATM*	c.8494C>T p.(Arg2832Cys)	c.8494C>T p.(Arg2832Cys)	c.7516-2A>G	c.6326G>A p.(Trp2109*)	-	
*BRCA1*	-	-	-	-	c.1121del p.(Thr374Asnfs*2)	-
*CHEK2*	-	-	-	-	-	c.499G>A p.(Gly167Arg)

ER—estrogen receptor; PR—progesterone receptor; SISH—silver in situ hybridization. Reference transcripts: *ATM* NM_000051.3, *BRCA1* NM_007294.3, *BRCA2* NM_000059.3, and *CHEK2* NM_007194.3. * refers to a stop codon.

**Table 3:** Characteristics of the variants identified in the patients.

Gene	Variant	Type	MAF	In Silico Predictors’ Results	ACMG Classification
*BRCA2*	c.3865_3868del p.(Lys1289Alafs*3)	Deletion	gnomAD: - ALFA: -	F: not applicable	Pathogenic
*BRCA2*	c.5057T>A p.(Leu1686*)	Nonsense	gnomAD: - ALFA: -	F: not applicable	Pathogenic
*BRCA2*	c.4284dup p.(Gln1429Serfs*9)	Duplication	gnomAD: 0.0004% ALFA: -	F: not applicable	Pathogenic
*BRCA2*	c.8243G>A p.(Gly2748Asp)	Missense	gnomAD: 0.0008% ALFA: 0.0039%	F: deleterious	Pathogenic
*BRCA2*	c.537dup p(Ile180Tyrfs*3)	Duplication	gnomAD: 0.0004% ALFA: -	F: not applicable	Pathogenic
*ATM*	c.8494C>T p.(Arg2832Cys)	Missense	gnomAD: 0.0030% ALFA: -	F: deleterious	Pathogenic
*ATM*	c.7516-2A>G	Splicing	gnomAD: - ALFA: -	F: deleterious HSF: Site acceptor broken	Likely pathogenic
*ATM*	c.6326G>A p.(Trp2109*)	Nonsense	gnomAD: - ALFA: -	F: not applicable	Pathogenic
*BRCA1*	c.1121del p.(Thr374Asnfs*2)	Deletion	gnomAD: - ALFA: -	F: not applicable	Pathogenic
*CHEK2*	c.499G>A p.(Gly167Arg)	Missense	gnomAD: 0.0024% ALFA: 0.0030%	F: deleterious	Pathogenic

F—Franklin by Genoox, MAF—Minor Allele Frequency, ACMG—American College of Medical Genetics and Genomics, HSF—Human Splicing Finder. Reference transcripts: *ATM* NM_000051.3, *BRCA1* NM_007294.3, *BRCA2* NM_000059.3, and *CHEK2* NM_007194.3.

## Data Availability

All the data relevant to the study is available in the manuscript.

## Supplementary Materials

The following supporting information can be downloaded at: https://www.mdpi.com/article/10.3390/jpm14060584/s1, Figure S1: Chromatograms of the variants identified in the patients; Table S1: DNA extraction; Table S2: Primer sequences used.

## References

[1] Espenschied C.R., LaDuca H., Li S., McFarland R., Gau C.-L., Hampel H. (2017). Multigene Panel Testing Provides a New Perspective on Lynch Syndrome. J. Clin. Oncol..

[2] Esplin E.D., Nielsen S.M., Bristow S.L., Garber J.E., Hampel H., Rana H.Q., Samadder N.J., Shore N.D., Nussbaum R.L. (2022). Universal Germline Genetic Testing for Hereditary Cancer Syndromes in Patients With Solid Tumor Cancer. JCO Precis. Oncol..

[3] Yadav S., Couch F.J. (2019). Germline Genetic Testing for Breast Cancer Risk: The Past, Present, and Future. Am. Soc. Clin. Oncol. Educ. Book..

[4] Rahman N. (2014). Mainstreaming genetic testing of cancer predisposition genes. Clin. Med..

[5] Whitaker K.D., Obeid E., Daly M.B., Hall M.J. (2021). Cascade Genetic Testing for Hereditary Cancer Risk: An Underutilized Tool for Cancer Prevention. JCO Precis. Oncol..

[6] Yap T.A., Ashok A., Stoll J., Mauer E., Nepomuceno V.M., Blackwell K.L., Garber J.E., Meric-Bernstam F. (2022). Prevalence of Germline Findings Among Tumors From Cancer Types Lacking Hereditary Testing Guidelines. JAMA Netw. Open.

[7] Kotsopoulos J., Hathaway C.A., Narod S.A., Teras L.R., Patel A.V., Hu C., Yadav S., Couch F.J., Tworoger S.S. (2023). Germline Mutations in 12 Genes and Risk of Ovarian Cancer in Three Population-Based Cohorts. Cancer Epidemiol. Biomark. Prev..

[8] Vietri M.T., Caliendo G., D’Elia G., Resse M., Casamassimi A., Minucci P.B., Dello Ioio C., Cioffi M., Molinari A.M. (2020). Five Italian Families with Two Mutations in BRCA Genes. Genes.

[9] Huang W., Bian J., Qian X., Shao L., Li H., Zhang L., Wang L. (2021). Case Report: Coinheritance of Germline Mutations in APC and BRCA1 in Colorectal Cancer. Front. Oncol..

[10] Andrés R., Menao S., Arruebo M., Quílez E., Cardiel M.J. (2019). Double heterozygous mutation in the BRCA1 and ATM genes involved in development of primary metachronous tumours: A case report. Breast Cancer Res. Treat..

[11] Slaught C., Berry E.G., Bacik L., Skalet A.H., Anadiotis G., Tuohy T., Leachman S.A. (2021). Clinical challenges in interpreting multiple pathogenic mutations in single patients. Hered. Cancer Clin. Pract..

[12] Lavie O., Narod S., Lejbkowicz F., Dishon S., Goldberg Y., Gemer O., Rennert G. (2011). Double heterozygosity in the BRCA1 and BRCA2 genes in the Jewish population. Ann. Oncol..

[13] Sokolenko A.P., Bogdanova N., Kluzniak W., Preobrazhenskaya E.V., Kuligina E.S., Iyevleva A.G., Aleksakhina S.N., Mitiushkina N.V., Gorodnova T.V., Bessonov A.A. (2014). Double heterozygotes among breast cancer patients analyzed for BRCA1, CHEK2, ATM, NBN/NBS1, and BLM germ-line mutations. Breast Cancer Res. Treat..

[14] Helleday T. (2010). Homologous recombination in cancer development, treatment and development of drug resistance. Carcinogenesis.

[15] Cortesi L., Piombino C., Toss A. (2021). Germline Mutations in Other Homologous Recombination Repair-Related Genes Than BRCA1/2: Predictive or Prognostic Factors?. J. Pers. Med..

[16] Chatterjee N., Walker G.C. (2017). Mechanisms of DNA damage, repair and mutagenesis. Environ. Mol. Mutagen..

[17] Khanna K.K. (2000). Cancer Risk and the ATM Gene: A Continuing Debate. JNCI J. Natl. Cancer Inst..

[18] Yamamoto H., Hirasawa A. (2021). Homologous Recombination Deficiencies and Hereditary Tumors. Int. J. Mol. Sci..

[19] Jonathan L., Philipp H., Charlie G., Michael F., Ignace V., Gordon R., Clare S., Werner M., Ronnie S.-F., Tamar S. (2012). Olaparib Maintenance Therapy in Platinum-Sensitive Relapsed Ovarian Cancer. N. Engl. J. Med..

[20] Moiseyenko V.M., Chubenko V.A., Moiseyenko F.V., Zhabina A.S., Gorodnova T.V., Komarov Y.I., Bogdanov A.A., Sokolenko A.P., Imyanitov E.N. (2014). Evidence for clinical efficacy of mitomycin C in heavily pretreated ovarian cancer patients carrying germ-line BRCA1 mutation. Med. Oncol..

[21] Conroy M., Borad M.J., Bryce A.H. (2017). Hypoxia-Activated Alkylating Agents in BRCA1-Mutant Ovarian Serous Carcinoma. Cureus.

[22] Alsop K., Fereday S., Meldrum C., deFazio A., Emmanuel C., George J., Dobrovic A., Birrer M.J., Webb P.M., Stewart C. (2012). BRCA Mutation Frequency and Patterns of Treatment Response in BRCA Mutation–Positive Women With Ovarian Cancer: A Report From the Australian Ovarian Cancer Study Group. J. Clin. Oncol..

[23] Cybulski C., Nazarali S., Narod S.A. (2014). Multiple primary cancers as a guide to heritability. Int. J. Cancer.

[24] Hall M.J., Bernhisel R., Hughes E., Larson K., Rosenthal E.T., Singh N.A., Lancaster J.M., Kurian A.W. (2021). Germline pathogenic variants in the Ataxia Telangiectasia Mutated (ATM) gene are associated with high and moderate risks for multiple cancers. Cancer Prev. Res..

[25] Harvengt J., Lumaka A., Fasquelle C., Caberg J.H., Mastouri M., Janssen A., Palmeira L., Bours V. (2023). HIDEA syndrome: A new case report highlighting similarities with ROHHAD syndrome. Front. Genet..

[26] Richards S., Aziz N., Bale S., Bick D., Das S., Gastier-Foster J., Grody W.W., Hegde M., Lyon E., Spector E. (2015). Standards and Guidelines for the Interpretation of Sequence Variants: A Joint Consensus Recommendation of the American College of Medical Genetics and Genomics and the Association for Molecular Pathology. Genet. Med..

[27] Desmet F.-O., Hamroun D., Lalande M., Collod-Béroud G., Claustres M., Béroud C. (2009). Human Splicing Finder: An online bioinformatics tool to predict splicing signals. Nucleic Acids Res..

[28] Ware M.D., DeSilva D., Sinilnikova O.M., Stoppa-Lyonnet D., Tavtigian S.V., Mazoyer S. (2006). Does nonsense-mediated mRNA decay explain the ovarian cancer cluster region of the BRCA2 gene?. Oncogene.

[29] Richardson M.E., Hu C., Lee K.Y., LaDuca H., Fulk K., Durda K.M., Deckman A.M., Goldgar D.E., Monteiro A.N.A., Gnanaolivu R. (2021). Strong functional data for pathogenicity or neutrality classify BRCA2 DNA-binding-domain variants of uncertain significance. Am. J. Hum. Genet..

[30] Guidugli L., Pankratz V.S., Singh N., Thompson J., Erding C.A., Engel C., Schmutzler R., Domchek S., Nathanson K., Radice P. (2013). A classification model for BRCA2 DNA binding domain missense variants based on homology directed repair activity. Cancer Res..

[31] Rebbeck T.R., Friebel T.M., Friedman E., Hamann U., Huo D., Kwong A., Olah E., Olopade O.I., Solano A.R., Teo S.-H. (2018). Mutational Spectrum in a Worldwide Study of 29,700 Families with BRCA1 or BRCA2 Mutations. Hum. Mutat..

[32] Laitman Y., Friebel T.M., Yannoukakos D., Fostira F., Konstantopoulou I., Figlioli G., Bonanni B., Manoukian S., Zuradelli M., Tondini C. (2019). The spectrum of BRCA1 and BRCA2 pathogenic sequence variants in Middle Eastern, North African, and South European countries. Hum. Mutat..

[33] Demir S., Tozkir H., Gurkan H., Atli E.I., Yalcintepe S., Atli E., Sezer A., Eker D., Tuncbilek N., Tastekin E. (2020). Genetic screening results of individuals with high risk BRCA- related breast/ovarian cancer in Trakya region of Turkey. J. BUON.

[34] Mitui M., Nahas S., Du L., Yang Z., Lai C., Nakamura K., Arroyo S., Scott S., Purayidom A., Concannon P. (2009). Functional and Computational Assessment of Missense Variants in the Ataxia-Telangiectasia Mutated (ATM) Gene: Mutations with Increased Cancer Risk. Hum. Mutat..

[35] Delia D., Mizutani S., Panigone S., Tagliabue E., Fontanella E., Asada M., Yamada T., Taya Y., Prudente S., Saviozzi S. (2000). ATM protein and p53-serine 15 phosphorylation in ataxia-telangiectasia (AT) patients and at heterozygotes. Br. J. Cancer.

[36] Cavalieri S., Pozzi E., Gatti R.A., Brusco A. (2013). Deep-intronic ATM mutation detected by genomic resequencing and corrected in vitro by antisense morpholino oligonucleotide (AMO). Eur. J. Hum. Genet..

[37] Borg Å., Haile R.W., Malone K.E., Capanu M., Diep A., Törngren T., Teraoka S., Begg C.B., Thomas D.C., Concannon P. (2010). Characterization of BRCA1 and BRCA2 Deleterious Mutations and Variants of Unknown Clinical Significance in Unilateral and Bilateral Breast Cancer: The WECARE Study. Hum. Mutat..

[38] Li J., Williams B.L., Haire L.F., Goldberg M., Wilker E., Durocher D., Yaffe M.B., Jackson S.P., Smerdon S.J. (2002). Structural and Functional Versatility of the FHA Domain in DNA-Damage Signaling by the Tumor Suppressor Kinase Chk2. Mol. Cell.

[39] Boonen R.A.C.M., Wiegant W.W., Celosse N., Vroling B., Heijl S., Kote-Jarai Z., Mijuskovic M., Cristea S., Solleveld-Westerink N., van Wezel T. (2022). Functional Analysis Identifies Damaging CHEK2 Missense Variants Associated with Increased Cancer Risk. Cancer Res..

[40] Cybulski C., Wokołorczyk D., Jakubowska A., Huzarski T., Byrski T., Gronwald J., Masojć B., Dębniak T., Górski B., Blecharz P. (2011). Risk of Breast Cancer in Women With a CHEK2 Mutation With and Without a Family History of Breast Cancer. J. Clin. Oncol..

[41] Couch F.J., Shimelis H., Hu C., Hart S.N., Polley E.C., Na J., Hallberg E., Moore R., Thomas A., Lilyquist J. (2017). Associations Between Cancer Predisposition Testing Panel Genes and Breast Cancer. JAMA Oncol..

[42] Kuchenbaecker K.B., Hopper J.L., Barnes D.R., Phillips K.-A., Mooij T.M., Roos-Blom M.-J., Jervis S., van Leeuwen F.E., Milne R.L., Andrieu N. (2017). Risks of Breast, Ovarian, and Contralateral Breast Cancer for BRCA1 and BRCA2 Mutation Carriers. JAMA.

[43] Graffeo R., Rana H.Q., Conforti F., Bonanni B., Cardoso M.J., Paluch-Shimon S., Pagani O., Goldhirsch A., Partridge A.H., Lambertini M. (2022). Moderate penetrance genes complicate genetic testing for breast cancer diagnosis: ATM, CHEK2, BARD1 and RAD51D. Breast.

[44] Levy-Lahad E., Friedman E. (2007). Cancer risks among BRCA1 and BRCA2 mutation carriers. Br. J. Cancer.

[45] Duzkale Teker N., Eyerci N. (2021). Double Heterozygous Mutations in the BRCA2 and ATM Genes: A Case Report and Review of the Literature. Breast Care.

[46] Maxwell K.N., Domchek S.M., Nathanson K.L., Robson M.E. (2016). Population Frequency of Germline BRCA1/2 Mutations. J. Clin. Oncol..

[47] Swift M., Morrell D., Cromartie E., Chamberlin A.R., Skolnick M.H., Bishop D.T. (1986). The incidence and gene frequency of ataxia-telangiectasia in the United States. Am. J. Hum. Genet..

[48] Nguyen-Dumont T., Dowty J.G., Steen J.A., Renault A.-L., Hammet F., Mahmoodi M., Theys D., Rewse A., Tsimiklis H., Winship I.M. (2021). Population-Based Estimates of the Age-Specific Cumulative Risk of Breast Cancer for Pathogenic Variants in CHEK2: Findings from the Australian Breast Cancer Family Registry. Cancers.

[49] Sukumar J., Kassem M., Agnese D., Pilarski R., Ramaswamy B., Sweet K., Sardesai S. (2021). Concurrent germline BRCA1, BRCA2, and CHEK2 pathogenic variants in hereditary breast cancer: A case series. Breast Cancer Res. Treat..

[50] Wang A., Everett J.N., Chun J., Cen C., Simeone D.M., Schnabel F. (2021). Impact of changing guidelines on genetic testing and surveillance recommendations in a contemporary cohort of breast cancer survivors with family history of pancreatic cancer. Sci. Rep..

[51] Hall M.J., McSweeny M.J., Rainey K., Campbell H., Nguyen C., Neumann C. (2023). Risks and implications of multiple actionable pathogenic germline variants discovered by panel-based cancer predisposition testing. J. Clin. Oncol..

[52] Weitzel J.N., Blazer K.R., Nehoray B., Kidd J., Slavin T.P., Solomon I., Niell-Swiller M., Rybak C., Saam J. (2015). Assessment of the clinical presentation of patients with at least two deleterious mutations on multi-gene panel testing. J. Clin. Oncol..

[53] Dal Buono A., Poliani L., Greco L., Bianchi P., Barile M., Giatti V., Bonifacio C., Carrara S., Malesci A., Laghi L. (2023). Prevalence of Germline Mutations in Cancer Predisposition Genes in Patients with Pancreatic Cancer or Suspected Related Hereditary Syndromes: Historical Prospective Analysis. Cancers.

