# Supplementary material for: Digenic Inheritance of Mutations in Homologous Recombination Genes in Cancer Patients
Source: J Pers Med. 2024 May 29;14(6):584. doi: 10.3390/jpm14060584 (PMC11204488; doi:10.3390/jpm14060584)
Supplement: Supplementary file 1 [file jpm-14-00584-s001.zip › jpm-2976998-supplementary.pdf]

Supplementary data

1. DNA extraction methods

STARlet

Germline DNA was extracted from 200 µl of peripheral blood using the NucleoMag Blood kit (Macherey-Nagel) according to the manufacturer's instructions. The NucleoMag® Blood 200 µL procedure is based on reversible adsorption of nucleic acids to paramagnetic beads under appropriate buffer conditions.

Finally, highly purified DNA was eluted with low-salt Elution Buffer MBL5 (100 µl) and directly used for downstream applications. The protocol was automated on a Starlet (Hamilton) robot.

QIAcube

Germline DNA was extracted from 200 µl of peripheral blood using the QIAamp DNA Blood Mini Kits (QIAGEN) according to the manufacturer's instructions. The QIAamp DNA Blood Kit provided silica-membrane-based DNA purification. During the DNA purification procedure, DNA bound specifically to the QIAamp silica-gel membrane while contaminants passed through. PCR inhibitors, such as divalent cations and proteins, were completely removed in two efficient wash steps, leaving pure DNA to be eluted in water (100 µl). The protocol was automated on a QiaCube (Qiagen) robot.

2. Genes´ accession numbers

Gene	SureMASTR	Twist
BRCA2	NM_000059.3	NM_000059.4
BRCA1	NM_007294.3	NM_007294.4
ATM	NM_000051.3	NM_000051.4
CHEK2	NM_007194.3	NM_007194.4

3. Software used

SeqPilot V4.3.1 (JSI, Ettenheim, GmbH, Germany) was used for Sanger and MLPA.

SeqPilot, module SeqNext v4.3 (JSI, Ettenheim, GmbH, Germany) was used for NGS with Agilent technologies.

ALISSA Interpret v5.4 (Agilent technologies, Santa Clara, CA, USA) was used for NGS with Twist Bioscience.

Table S1. DNA extraction.

	**Patient 1**		**Patient 2**	**Patient 3**		**Patient 4**	**Patient 5**		**Patient 6**	
	Sample 1	Sample 2	Sample 1	Sample 1	Sample 2	Sample 1	Sample 1	Sample 2	Sample 1	Sample 2
Type of sample	Blood	Blood	Blood	Blood	Blood	Blood	Blood	Blood	Blood	Blood
DNA extraction method	QIAcube	STARlet	QIAcube	STARlet	QIAcube	QIAcube	QIAcube	STARlet	STARlet	QIAcube
Spectroscopic analysis	NanoDrop	NanoDrop	NanoDrop	NanoDrop	NanoDrop	NanoDrop	NanoDrop	NanoDrop	NanoDrop	NanoDrop
Purity (260/280 ratio)	1.62	2.00	1.80	1.98	1.77	1.62	1.79	2.13	1.91	1.71
Concentration (ng/ul)	25.45	67.76	52.95	208.98	89.13	43.36	56.70	95.46	80.45	50.58
Sequencing kit	SureMASTR Hereditary Cancer Agilent kit, 26 genes	Sanger	SureMASTR Hereditary Cancer Agilent kit, 12 genes + MLPA.<br>Sanger	SureMASTR Hereditary Cancer Agilent kit, 12 genes + MLPA	Sanger	SureMASTR Hereditary Cancer Agilent kit, 12 genes + MLPA.<br>Sanger	High-throughput Breast cancer panel 26 genes + MLPA	Sanger	13-gene Breast cancer panel + MLPA	Sanger

Panel manufacturer	Agilent technologies, Santa Clara, CA, USA	-	Agilent technologies, Santa Clara, CA, USA	Agilent technologies, Santa Clara, CA, USA	-	Agilent technologies, Santa Clara, CA, USA	Twist Bioscience, San Francisco, CA, USA)	-	Twist Bioscience, San Francisco, CA, USA)	-
Sequenced genes	*BRCA1, BRCA2, PALB2, CHEK2, BARD1, BRIP1, RAD51C, RAD51D, TP53, MRE11A, RAD50, NBN, FAM175A, ATM, STK11, MEN1, PTEN, CDH1, MUTYH, BLM, XRCC2, MLH1, MSH6, PMS2* and *MSH2* and the 3' UTR of *EPCAM*	*BRCA2, ATM*	*BRCA1, BRCA2, PALB2, TP53, CHEK2, MLH1, MSH2, MSH6, ATM, BRIP1, RAD51C and RAD51D*	*BRCA1, BRCA2, PALB2, TP53, CHEK2, MLH1, MSH2, MSH6, ATM, BRIP1, RAD51C and RAD51D*	*BRCA2, ATM*	*BRCA1, BRCA2, PALB2, TP53, CHEK2, MLH1, MSH2, MSH6, ATM, BRIP1, RAD51C and RAD51D*	*BRCA1, BRCA2, PALB2, TP53, CHEK2, CDH1, STK11, PTEN, MLH1, MSH2, MSH6, MTYH, EPCAM, MEN1, BRIP1, RAD51C, RAD51D, ATM, BARD1, RAD50, BLM, FAM175A, MRE11A, NBN,*	*BRCA1, BRCA2*	*BRCA1, BRCA2, PALB2, TP53, CHEK2, MLH1, MSH2, MSH6, ATM, BRIP1, RAD51C, RAD51D, BARD1*	BRCA2, *CHEK2*

							XRCC2, PMS2			
MLPA	-	-	*BRCA1, BRCA2*	*BRCA1, BRCA2*	-	*BRCA1, BRCA2*	*BRCA1, BRCA2, MLH1, MSH2, MSH6*	-	*BRCA1, BRCA2, CHEK2* c.1100del	-
Amplicon or enrichment method	Amplicon	-	Amplicon	Amplicon	-	Amplicon	Enrichment method	-	enrichment method	-
Reference genome	hg19	hg19	hg19	hg19	hg19	hg19	hg19	hg19	hg19	hg19
Bioinformatic pipeline	Inhouse demultiplexing pipeline	-	Inhouse demultiplexing pipeline	Inhouse demultiplexing pipeline	-	Inhouse demultiplexing pipeline	Inhouse Humanomics pipeline	-	Inhouse Humanomics pipeline	-

Table S2. Primer sequences used.

Patient	Gene	Exon	Forward primer sequence	Reverse primer sequence
Patient 1	*BRCA2*	11-05	AAGTGCCTGAAAACCAGATG	CAACAAAAGTGCCAGTAGTCA
	ATM	58	GCTTCCCTGTCCAGACTGTT	CACTATCATCCCCCTGCAAC
Patient 2	*BRCA2*	11-05	AAGTGCCTGAAAACCAGATG	CAACAAAAGTGCCAGTAGTCA
	ATM	58	GCTTCCCTGTCCAGACTGTT	CACTATCATCCCCCTGCAAC
Patient 3	*BRCA2*	11-08	TGAGACCATTGAGATCACAGC	TAGTCACAAGTTCCTCAACGCA
	ATM	51	TGCATTAATCTAGAGTACCCATTAG	GAAATCCTAGGCCTCCCA
Patient 4	*BRCA2*	11-07	TTGTCAGATTTAACTTTTTTGGAAG	CAACTGGGACACTTTCTTTCAG
	ATM	43	TCAAACTCCTGGGCTCAAGT	CAGTTGTTGTTTAGAATGAGGAGAGA
Patient 5	*BRCA1*	10.3-4 (11-03)	GGGCTGGAAGTAAGGAAACAT	ACGCTCTTGTATTATCTGTGG

	BRCA2	18	CAGTGGAATTCTAGAGTCAC	GAAAGATCTCTGGACCTCC
Patient 6	*BRCA2*	7	GCAATTCAGTAAACGTTAAGTG	GTCAGTTACTAACACACTTATC
	CHEK2	4	GGAGAGCTGGTAATTTGGTCA	CGCCTCAGCCTCCCAAAG

Figure S1. Chromatograms of the variants identified in the patients.

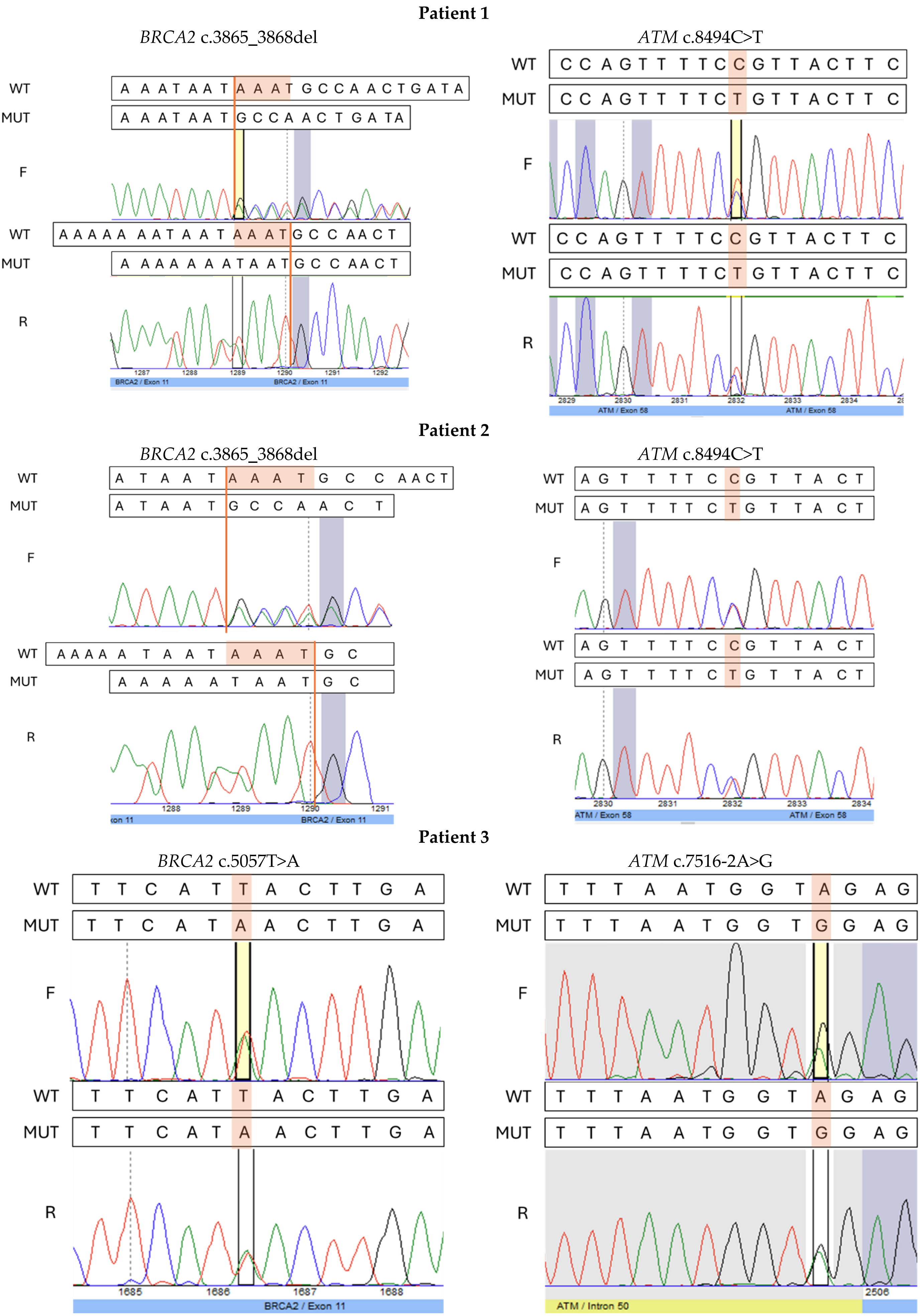

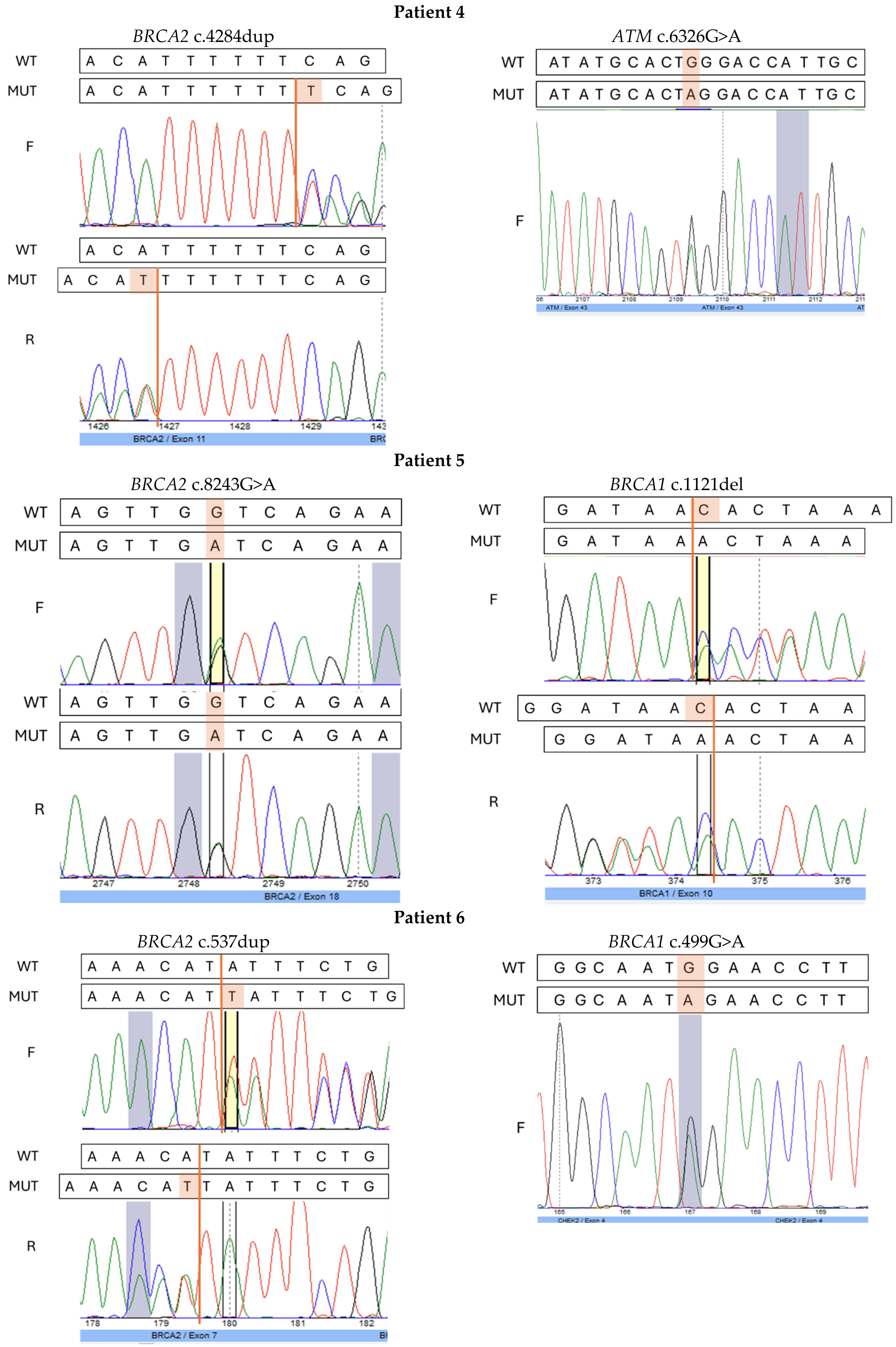

F – forward primer, MUT – Mutant, R – reverse primer WT – wild type
